# A Machine Learning‐Guided Search of Cu‐Based Bimetallic Alloys That Favor CO Dimerization in CORR

**DOI:** 10.1002/cssc.202501603

**Published:** 2026-03-26

**Authors:** Mattia Salomone, Wei Wang, Federico Raffone, Michele Re Fiorentin, Francesca Risplendi, Giancarlo Cicero

**Affiliations:** ^1^ Department of Applied Science and Technology Politecnico di Torino Torino Italy; ^2^ Nano‐Bio Spectroscopy Group, European Theoretical Spectroscopy Facility (ETSF), Department of Advanced Materials and Polymers: Physics, Chemistry and Technology University of the Basque Country UPV/EHU San Sebastián Spain

**Keywords:** C_2_ products, CO_2_RR, Cu‐based catalysts, high throughput screening, machine learning

## Abstract

The rational design of Cu‐based bimetallic catalysts for the electrochemical reduction of CO_2_ into multicarbon (C_2_/C_2+_) products critically depends on tuning the CO adsorption strength, which governs C–C coupling selectivity. However, systematically exploring the vast configurational space of CuM alloys through density functional theory (DFT) is computationally prohibitive. We developed a machine learning (ML) framework to predict CO adsorption behavior on Cu‐based bimetallic surfaces using a physically interpretable feature set, including geometric descriptors (nearest‐neighbor distance, coordination number) and elemental properties (electronegativity, ionization energy), which collectively determine the local electronic environment of the CO binding sites. A two‐step ML protocol, combining a Gradient Boosting Classifier to identify stable adsorption sites and a Gradient Boosting Regressor to predict their adsorption energies, was trained on DFT data for 15 CuM(111) and CuM(100) systems, resulting in a total of 1,515 structures. The models were then applied to screen 29 CuM alloys, encompassing about 91,000 adsorption sites across multiple surface concentrations and configurations of the alloying atoms. The screening identified CuAg, CuAl, CuAu, CuZn, CuIn, and CuGa as promising candidates for promoting CO–CO coupling, the rate‐determining step toward C_2_ product formation. Among these, CuGa was selected for further validation as a representative system, having received far less attention in the CO_2_RR literature than other well‐studied alloys (CuAg, CuAl, and CuAu). Constant‐potential DFT calculations confirmed the ML predictions, revealing that CO dimerization on CuGa(100) proceeds with a more favorable reaction energy and an activation barrier about 0.2 eV lower than on pure Cu(100).

## Graphical abstract

A machine learning–driven screening identifies Cu‐based bimetallic alloys that enhance CO–CO coupling in CO_2_ electroreduction. Using interpretable geometric and elemental features, the framework predicts CO adsorption behavior across thousands of sites, highlighting CuGa, CuAg, CuAl, CuAu, CuZn, and CuIn as promising catalysts for selective C_2_ formation.

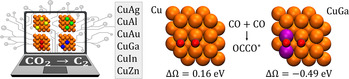

## 1 Introduction

The urgent need to mitigate anthropogenic climate change has driven the development of technologies for CO_2_ capture and conversion into valuable chemicals [1]. Among these, electrochemical CO_2_ reduction (CO_2_RR) powered by renewable energy has emerged as a particularly promising approach [2]. While significant progress has been made in the selective production of single‐carbon (C_1_) products such as CO and formic acid [3, 4], the efficient and selective conversion of CO_2_ into multicarbon (C_2+_) products—such as ethylene and ethanol, which have higher market potential—remains challenging. This difficulty arises from the inherently low selectivity toward C_2+_ species, largely due to competing pathways such as hydrogen evolution, which is especially dominant under the acidic conditions required for appreciable CO_2_ solubility [5]. Conversely, while alkaline environments suppress HER, CO_2_ becomes scarce, creating a fundamental trade‐off. To date, no catalyst has demonstrated both high selectivity and efficiency for direct CO_2_‐to‐C_2+_ conversion. Copper is unique among metallic catalysts for its ability to produce C_2+_ molecules. On Cu surfaces, CO_2_ is initially reduced to CO [6], which remains adsorbed and can undergo CO–CO dimerization, a process that is widely recognized as the rate‐limiting step in the formation of C_2_ and higher‐order carbon products [7, 8]. This mechanistic insight has motivated the development of the tandem strategy [9, 10], in which CO_2_ is first reduced to CO using catalysts such as Ag, Au, or Zn [11, 12, 13], or other highly efficient materials [14, 15] achieving selectivity larger than 95%, and subsequently fed into a second reactor, where Cu‐based catalysts convert CO into C_2+_ products under alkaline conditions. These media suppress the competing hydrogen evolution reaction (HER) and provide a favorable environment for C–C coupling [16, 17]. Despite this advancement, pure copper still suffers from limited CO dimerization activity, restricting overall C_2_‐product selectivity and productivity. These limitations have motivated extensive efforts to engineer the surface properties of Cu catalysts through alloying and structural modification. Due to the critical role of CO as a reactive intermediate [18, 19, 20, 21, 22, 23], a key design strategy is to tune its binding energy, which strongly influences the feasibility of the dimerization step. According to the Sabatier principle [24], CO must bind neither too weakly (leading to desorption) nor too strongly (leading to surface poisoning) [25, 26, 27]. Understanding how this balance manifests under relevant operating conditions is therefore essential. In the experimental configuration considered here, i.e., a tandem reactor where CO is generated upstream with > 95% selectivity and C–C coupling occurs under alkaline conditions, the CO adsorption energy, already recognized as a key descriptor for CO_2_ and CO electroreduction, emerges as the most relevant parameter for assessing the propensity of Cu‐based surfaces to promote C–C coupling. Under these conditions, the mechanistic bottlenecks shift compared to conventional CO_2_ reduction: the CO_2_ activation barrier no longer enters the reaction kinetics, the hydrogen evolution reaction is strongly suppressed, and OH adsorption is thermodynamically unfavorable [28]. As a result, CO species dominate the reactive surface, and their adsorption strength dictates the likelihood of initiating C–C coupling. Among various strategies proposed to modulate the CO binding strength, alloying Cu with a second metal (CuM) has shown particular promise for enhancing both catalytic activity and selectivity [29, 30, 31, 32, 33, 34, 35]. However, the enormous compositional and structural space of bimetallic alloys makes the identification of optimal CuM combinations extremely challenging. Density functional theory (DFT) has been instrumental in providing atomic‐scale insights into adsorption thermodynamics and reaction mechanisms for CO_2_RR catalysts [36, 37, 38]. However, its high computational cost makes a systematic exploration of the vast compositional and configurational space of Cu‐based bimetallic alloys practically unfeasible. The situation becomes even more complex when considering different surface facets, impurity types, concentrations, and arrangements [39, 40, 41, 42]. As a result, most existing studies are limited to a handful of alloy compositions or to specific adsorption sites, providing only a partial picture of the catalytic landscape. To overcome these limitations, combining DFT with machine learning (ML) models offers a powerful strategy for screening large materials spaces with significantly reduced computational cost, enabling the rapid identification of promising catalyst candidates. By training models on DFT‐derived data, ML enables rapid predictions of adsorption properties across unexplored materials, allowing for large‐scale screening with orders‐of‐magnitude lower computational demand. Once trained, such models can generalize to unseen alloy configurations and compositions, making them particularly suitable for rational catalyst discovery [43, 44, 45, 46, 47]. ML has already shown success in various catalytic systems [25, 48, 49, 50, 51, 52, 53, 54], but despite growing interest in Cu‐based bimetallic catalysts for CO_2_RR, a dedicated ML‐driven screening specifically aimed at boosting C_2+_ product formation through enhanced CO dimerization has been lacking. This gap is particularly relevant given the central role of CO–CO coupling in determining C_2_‐product selectivity and the vast combinatorial space of possible CuM alloy configurations.

In this study, we build on the ML‐based strategy introduced in our previous work [55] to close this gap. We enhance the modeling framework by training new models on a broader and more representative dataset, including additional geometric and chemical descriptors that improve both accuracy and generalizability. The expanded dataset and feature space allow the models to better capture the diversity of adsorption environments characteristic of bimetallic systems, where local composition and geometry can vary significantly even at fixed stoichiometry. The workflow follows a two‐step ML protocol: a classification stage to identify stable CO adsorption sites and a regression stage to predict their corresponding adsorption energies ($\Delta E_{\mathrm{CO}}$) [56, 57]. This hierarchical scheme ensures that the computational effort is focused on physically meaningful adsorption configurations while retaining high predictive accuracy. The feature set integrates tabulated elemental properties with structural descriptors of the local adsorption environment, all selected to be readily available without additional DFT calculations. This choice ensures full decoupling between feature construction and explicit simulations, making the proposed framework scalable to thousands of candidate structures. We apply this method to conduct a high‐throughput screening of the (111) and (100) facets of Cu‐based bimetallic alloys, analyzing a wide variety of alloying atoms, concentrations, and configurations. To evaluate the propensity of these surfaces to promote C–C coupling, we then define screening criteria to identify pairs of CO adsorption sites which are more likely to promote CO dimerization than those on pure Cu. In particular, here we focus on CO dimerization on the Cu(100) surface, where it has been shown that the reaction proceeds from two CO molecules adsorbed on facing bridge sites and producing the resulting dimer in the middle hollow site [58, 59, 60]. Our proposed screening criteria rely on both thermodynamic and geometric considerations, reflecting the need for optimal binding strength as well as favorable spatial arrangement for CO–CO coupling. The alloys that consistently meet these criteria are highlighted as promising candidates for CORR. Based on this analysis, CuAg, CuAl, CuAu, CuZn, CuGa, and CuIn emerged as the most promising alloys among those evaluated. Notably, the recurrence of specific alloying elements across different surface facets underscores their robust ability to modulate CO adsorption in a manner conducive to C–C bond formation. Among these, CuGa was selected for further validation because, unlike CuAg, CuAl, or CuAu, it remains comparatively unexplored in the context of CO2RR. This choice allows us to both validate the predictions of our ML‐based screening framework and provide new insights into a less‐studied, yet promising, Cu‐based alloy system. Constant‐potential DFT calculations confirmed its potential to enhance C–C coupling in CORR, supporting the robustness and predictive power of the proposed approach.

## 2 Method

ML models were trained on DFT‐calculated CO adsorption energies (considering C as the atom binding to the surface) on Cu‐based bimetallic alloys. All DFT calculations were performed with the Quantum Espresso code [61, 62], using ultrasoft pseudopotentials [63] and the Perdew–Burke‐Ernzerhof (PBE) exchange‐correlation functional [55, 64, 65].

Valence electron wavefunctions were expanded using plane waves with a kinetic‐energy cutoff of 40 Ry, while for the charge density we employed a cutoff of 400 Ry. Pristine copper surfaces were represented by four‐layered Cu(111)/(100) (1×1) slabs with a 12 Å vacuum layer in each supercell. The bottom two layers were fixed, while the topmost were relaxed by minimizing the atomic forces with a convergence threshold of $10^{-5}$ Ry/Bohr. The Brillouin zone was sampled using a 10×10×1 Monkhorst–Pack k‐point grid [66].

**FIGURE 1:**
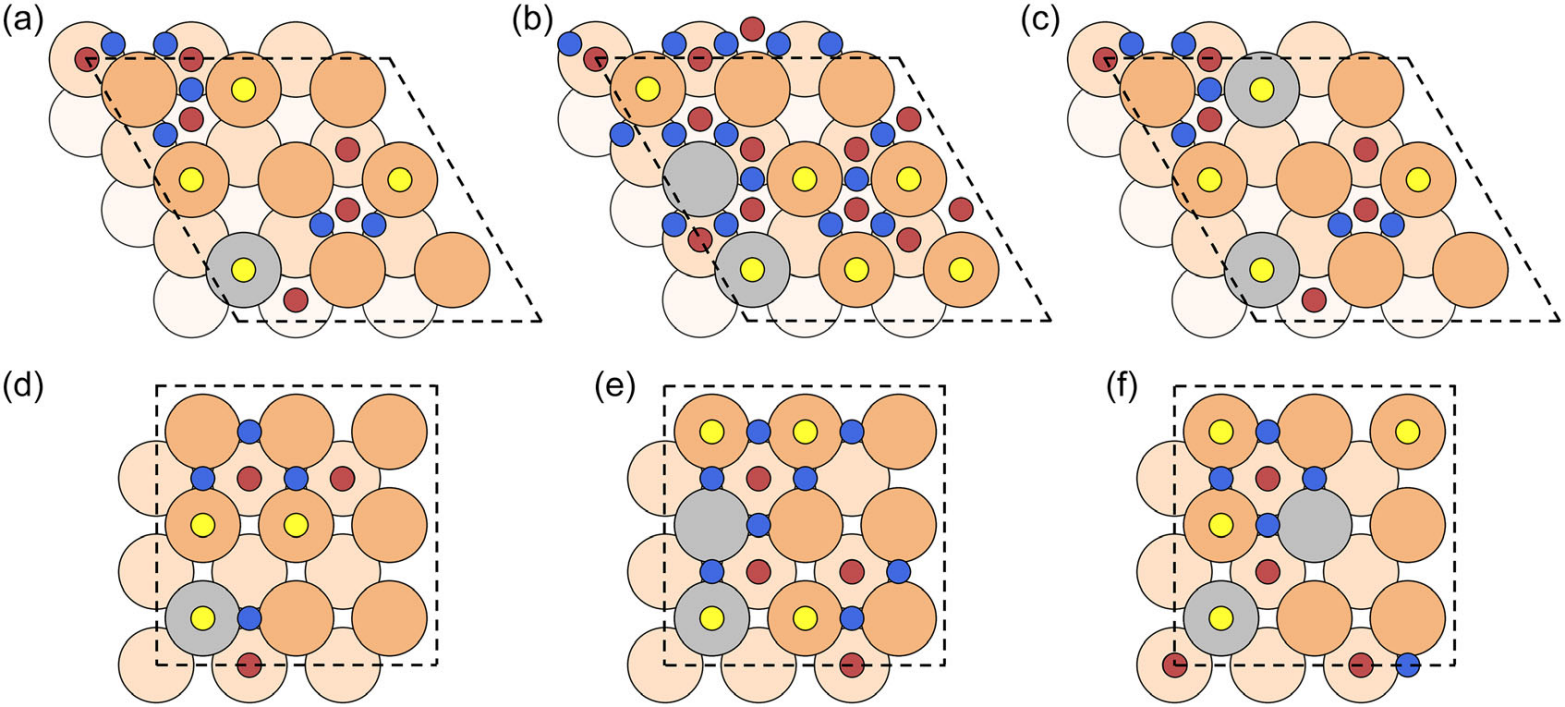
Surface alloy structures used to build the DFT dataset for training the ML models. Panels (a) and (d) show configurations containing a single alloy atom, designed to provide examples of CO adsorption in the dilute‐guest species regime. Panels (b), (c), (e), and (f) contain multiple alloy atoms placed in first‐ and second‐neighbor positions (considering periodic boundary conditions) to allow the dataset to capture how CO adsorption changes when several alloying atoms are located in close proximity. Black dashed lines indicate the unit cells. Orange and gray circles denote Cu and M atoms, respectively, while the smaller yellow (Top), blue (Bridge), and red (Hollow) circles mark the unique adsorption sites considered in the dataset.

To build the dataset of CO adsorption energies on different copper alloy surfaces, we built Cu(111)/(100)(3×3) surface supercells and substituted one or two Cu atoms of the surface layer with an alloying metal atom M. This corresponds to an alloy with a stoichiometry Cu_1−*x*_M_*x*_, where x=0.11 and 0.22 respectively. Fifteen different M species were included in the dataset, namely: Ag, Al, Au, Cd, Co, Fe, Hf, Ir, Mo, Nb, Ni, Pd, Pt, Ti, and V. The surface structures are shown in Figure 1.

The dataset labels correspond to the CO adsorption energies on all the inequivalent binding sites in each supercell,

$$\Delta E_{\mathrm{CO}}=E_{\mathrm{slab}+\mathrm{CO}}-E_{\mathrm{slab}}-E_{\mathrm{CO}}$$

where $E_{\mathrm{slab}+\mathrm{CO}}$, $E_{\mathrm{slab}}$, and $E_{\mathrm{CO}}$ are the energies of the Cu_1−*x*_M_*x*_ slab with adsorbed CO, the clean Cu_1−*x*_M_*x*_ surface, and an isolated CO molecule, respectively. The orange and gray circles in Figure 1 indicate the Cu and M atoms, while the smaller yellow (Top), blue (Bridge), and red (Hollow) circles mark the unique adsorption sites.

To ensure effective generalization of the ML model across different alloying‐atom configurations, we selected surface structures that exhibit representative examples of possible CuM arrangements. At the same time, to minimize surface reconstruction effects, which are beyond the scope of the present ML model, we limited the alloying‐atom concentration to x=0.22. Figure 1a,d show structures where the M atoms are sufficiently spaced to represent isolated guest species, as observed in our previous work [55], thus describing CO adsorption in the dilute‐alloy regime. In contrast, Figure 1b,c,d,f provide examples of alloying‐atom clustering, with M atoms arranged at nearest‐ and next‐to‐nearest‐neighbor distances, enabling the dataset to capture how CO adsorption changes when multiple guest species are in close proximity. Considering the inequivalent adsorption sites across each surface structure and alloying atoms, the final dataset comprises a total of 1515 labeled data points. Each label is uniquely identified by a vector of 21 features: 13 associated with the chemical properties of the guest species and 8 describing the geometry of the CO binding site. To develop an interpretable, general, accessible, and computationally efficient ML model, the chemical features are chosen to be readily available in online databases [67, 68, 69] without requiring additional DFT simulations. They include the atomic group, mass, period, and radius (AtGroup, AtMass, AtPer, AtRad); boiling and melting points (BoilPoint, MeltPoint); dipole polarizability (DipPol); electron affinity (ElAff); Pauling electronegativity (EnPaul); first ionization energy (IonEn); lattice parameter of the guest material in its stable bulk phase (LatPar); and the surface energy (SurfEn) and work function (WorkFun) of the hexagonal surface of the guest atom in its crystalline phase. The 8 geometrical features that describe the local environment of the CO binding site are as follows: the generalized coordination number [70] (GCN), the minimum distance from an alloying atom (minD), the number of Cu and guest species in the nearest‐ and next‐to‐nearest‐neighbor shells (n1h, n1g for the first; n2h, n2g for the second), and two features characterizing arrangement symmetries of the M atoms based on their geometric center of mass (dcm1 and dcm2). Further details are provided in Paragraph 3 of the Supporting Information (Figure S3). For ML model training and hyperparameter optimization, we used the *GridSearchCV* function of Scikit‐Learn [71] with 8‐fold cross‐validation [72]. In this procedure, the algorithm systematically tests all the possible combinations of hyperparameters within predefined ranges and selects the configuration that provides the best overall performance while avoiding overfitting. This approach ensures a balanced trade‐off between model accuracy and generalization. The dataset was split into 80% for training and 20% for testing. To ensure a well‐balanced test set, we employed *StratifiedShuffleSplit*, which maintained an approximately equal number of data points for each guest atom, preventing biases in the distribution.

The reaction energies of CO dimerization processes were determined using DFT simulations at constant‐potential on a four‐layer‐thick Cu(100) slab, with a 4×4 periodicity. Employing ENVIRON [62, 73], we used solvent‐aware solvation models with an explicit water layer of 10 H_2_O molecules (whose structure is described in Paragraph 1, Figure S1, of the Supporting Information) and implicit solvation. The electronic grand canonical energies Ω of initial and final geometries at constant potential U were calculated as [74, 75, 76]

$$\Omega =E_{\mathrm{DFT}}-N_{e}\mu_{e}(U)$$

where $E_{\mathrm{DFT}}$ is the DFT‐obtained electronic energy, $N_{e}$ is the number of added/removed electrons, and $\mu_{e}$ is the electronic chemical potential (referenced to the SHE scale as $\mu_{e}=-\Phi_{\mathrm{SHE}}-U$, with $\Phi_{\mathrm{SHE}}=4.44$ V [77]). For the activation barrier calculation, the transition state was identified at constant potential using the dimer method [78], with the initial guess obtained with the nudged elastic band method [79, 80] at constant charge.

## 3 Results and Discussion

### 3.1 Performance of the Machine Learning Models

With the dataset assembled and a comprehensive set of features defined, we proceeded to train and evaluate a series of machine learning models. This step is critical not only to assess the predictive power of the models but also to ensure their generalizability across different alloy compositions and surface configurations. In the following section, we benchmark the performance of several classification and regression algorithms to identify which are most effective for predicting CO adsorption behavior, an essential prerequisite for the high‐throughput screening effort described later in the paper. Different algorithms were tested to identify those best suited to our dataset, in line with the idea that no single model performs optimally across all tasks and data distributions [81]. Our strategy to describe the CO‐surface adsorption on the CuM catalyst surface is twofold. First, classification models are used to distinguish between stable binding sites, where CO adsorption is favorable, and unstable ones, where CO is repelled. Second, regression models are employed to predict the adsorption energy on the stable sites. Accordingly, we assessed both classification and regression algorithms with the optimized hyperparameters reported in Table 1. Specifically, we tested Logistic Regression (LRC) and Linear Regression (LR) [82], Decision Tree Classifier (DTC) and Regressor (DTR) [83], Random Forest Classifier (RFC) and Regressor (RFR) [84], Gradient Boosting Classifier (GBC) and Regressor (GBR) [85, 86], and Support Vector Machines for classification (SVC) and regression (SVR) [87].

**TABLE 1:** Optimized hyperparameters for the classification (upper table) and regression (lower table) models. An 8‐fold cross validation has been used.

Class.	Hyperparameters
Model	
LRC	C = 8
DTC	max_depth = 10, max_features = 14
	max_leaf_nodes = 73
	min_samples_split = 3
RFC	n_estimators = 10, max_depth = 12
	max_features = 5
	min_samples_leaf = 2
	max_samples = 0.8
GBC	n_estimators = 52, max_depth = 6
	learning_rate = 0.125
	max_features = 7
	min_samples_leaf = 6
	min_samples_split = 15
SVC	kernel = poly, coef0 = 0.1, C = 0.359
	gamma = 0.149
**Reg.**	
**Model**	**Hyperparameters**
LR	Default
DTR	criterion = squared_error
	max_depth = 7, max_leaf_nodes = 61
	min_samples_leaf = 2
RFR	bootstrap = False, n_estimators = 34
	max_depth = 10, max_features = 16
GBR	n_estimators = 25, max_depth = 5
	learning_rate = 0.17
	max_features = 15
SVR	kernel = poly, coef0 = 0.89, C = 0.135
	gamma = 0.125

After completing hyperparameter optimization, we evaluated the model performance on a test set comprising 20% of the available data. For classification models, performance was evaluated using the weighted average F1 score (Table 2), while the regression models were assessed using the Root Mean Squared Error (RMSE) and the coefficient of determination R2 (Table 3) [88, 89].

**TABLE 2:** F1 scores for the different ML algorithms used on the training (left column) and test (right column) sets.

Classification algorithm	Train	Test
LRC	0.80	0.77
DTC	0.95	0.92
RFC	0.94	0.88
GBC	0.96	0.90
SVC	0.94	0.90

**TABLE 3:** R2 score and RMSE for the different ML models on the training (left columns) and test (right columns) sets.

Regression	$R^{2}$		RMSE, eV	
Algorithm	Train	Test	Train	Test
LR	0.57	0.50	0.33	0.31
DTR	0.98	0.95	0.06	0.10
RFR	1.00	0.96	0.03	0.09
GBR	0.99	0.96	0.04	0.08
SVR	0.96	0.94	0.10	0.10

With the exception of LRC, all classification models achieved high predictive accuracy, as shown in Table 2. Despite minor differences in F1 scores, the results were generally consistent across training and test sets, indicating good generalizability. Given the overall reliability of the models, further comparison is needed to identify the most suitable one for our specific application.

A similar trend is observed for the regression task, as shown in Table 3. The simple LR model fails to provide accurate predictions, while all other models exhibit high predictive performance, with consistently comparable R2 scores and RMSE values.

To further evaluate the generalizability of our models (excluding LRC and LR due to their significantly lower performance), we conducted two additional tests, independently examining the impact of variations in chemical and geometric features.

The first test set (hereafter referred to as TsC) was designed to evaluate the ability of the models to extrapolate to unseen alloying elements while maintaining the geometric environment consistent with the training data. Specifically, we considered structures with spatially isolated guest species at a surface concentration of approximately 2.8% (Paragraph 4 of the Supporting Information, Figure S4), introducing new M atoms, namely Ru, W, and Zn, not present in the training set. The second test set (TsG) probes model performance under varying geometric configurations, while restricting the alloying elements to those included during training. For this test, we selected a representative subset of guest species (Ag, Al, Au, Fe, Ni, Pd, and Pt) to ensure a broad sampling of both stable and unstable adsorption sites, as well as a wide range of adsorption energies. In TsG, we introduced new M‐atom arrangements and concentrations to systematically explore geometric variability (Paragraph 4 of the Supporting Information, Figure S5).

**FIGURE 2:**
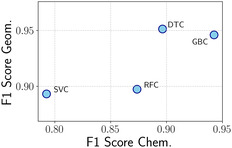
F1 scores achieved on TsC (*x*‐axis) and those obtained on TsG (*y*‐axis).

**FIGURE 3:**
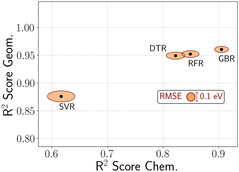
Performance of regression models on the TsC and TsG test sets. Each black dot represents a model, with its coordinates corresponding to the R^2^ scores on TsC (*x*‐axis) and TsG (*y*‐axis). The orange ellipses indicate the associated RMSE values where the width and height reflect the RMSE on TsC and TsG, respectively.

The classification results are reported in Figure 2, where the *x*‐axis denotes the F1 scores obtained on the TsC dataset, while the *y*‐axis shows those achieved on TsG. All models demonstrate strong performance in the second case, which includes geometric variations, with DTC and GBC slightly outperforming RFC and SVC. In contrast, when generalizing to unseen alloying elements, GBC displays a marked advantage in predictive accuracy, achieving an F1 score of 0.94. This substantially exceeds the performance of DTC (0.90), RFC (0.87), and SVC (0.79). Given its superior ability to generalize across different chemical compositions, GBC was selected as the classification model for high‐throughput screening. Regarding the regression task, we report the obtained results in Figure 3, following the same approach as before: the *x*‐axis reports metrics on the TsC dataset, while the *y*‐axis reflects performance on TsG. Each black dot represents a regression model, with its coordinates (*x*,*y*) corresponding to the respective R^2^ scores on TsC and TsG. The associated RMSE values are depicted as orange ellipses (centered on the corresponding black dot), whose width and height reflect the RMSE values for TsC and TsG, respectively. Consistently with the classification results, all models exhibit stronger generalization when novel geometric configurations are considered, achieving R^2^ values exceeding 0.85 and RMSEs at or below 0.1 eV—particularly for GBR, DTR, and RFR, while SVR performs comparably but with slightly higher errors. On the other hand, model accuracy decreases when the test set includes previously unseen alloying elements within the Cu host matrix, with SVR showing the most pronounced drop in performance. Among all evaluated models, GBR demonstrates the best overall performance. While its predictive accuracy is somewhat reduced on TsC relative to TsG, it still achieves a high R^2^ score of approximately 0.91 and an RMSE of 0.16 eV—twice the error observed on TsG, yet still within an acceptable range for practical applications.

**FIGURE 4:**
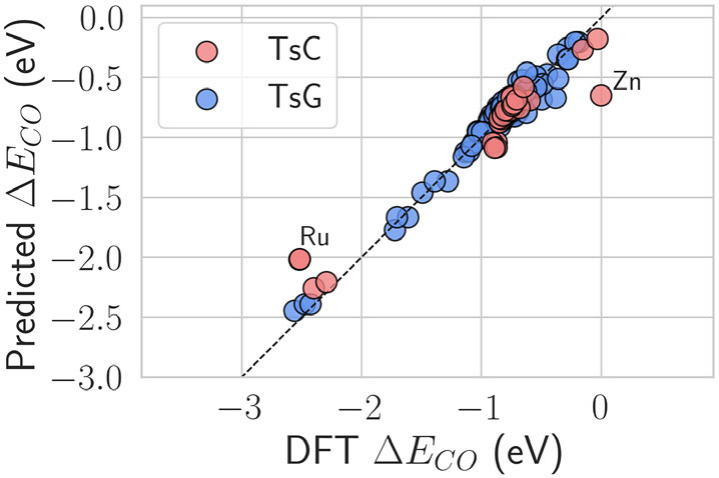
Parity plots obtained with the GBR for TsC (red circles) and TsG (blue circles). For TsG, the predictions are accurate across the whole CO adsorption energy range, while for TsC we observe some relevant deviations for extreme $\Delta E_{CO}$ values, as in the case of Ru and Zn, marked in the plot.

To further evaluate the reliability of the GBR model across different alloying elements and M‐atom configurations, we present a parity plot (see Figure 4) comparing predicted and DFT‐calculated CO adsorption energies. An analysis of the trend shows that the increased prediction error on TsC primarily arises from configurations with either low or high adsorption energy values. While GBR accurately predicts ΔECO for the majority of binding sites in TsG (blue circles), it tends to underestimate (overestimate) highly (slightly) negative values for TsC, as highlighted by the red circles that deviate most from the dashed line in Figure 4. For instance, in the case of CO adsorbed on top of Ru (marked in Figure 4) where DFT calculations yield adsorption energy around −2.5 eV, GBR predicts a less negative value of around −2.0 eV. Conversely, for CO on top of Zn (highlighted in Figure 4), the model overestimates the adsorption strength, returning an adsorption energy of approximately −0.6 eV compared to the DFT outcome of −0.03 eV. We rationalize these discrepancies as a consequence of the absence of features explicitly describing the CO adsorption orientation. In all cases showing larger prediction errors, the adsorbed CO molecule exhibited a marked tilt with respect to the surface normal. Since the current feature set only includes descriptors derived from idealized adsorbate–slab geometries, these deviations reflect a limitation inherent to the present framework. Nevertheless, such deviations, while slightly affecting the global error metrics, do not compromise the practical applicability of the model, which reliably captures the key adsorption trends across different systems. In the context of high‐throughput screening, where thousands of candidate structures must be evaluated efficiently, correctly identifying the adsorption strength regime is often more critical than achieving chemical accuracy for every individual case. Within this framework, the performance of the model is adequate to guide the early stages of catalyst discovery.

Given their ability in capturing CO adsorption trends, GBC and GBR emerge as the most suitable models for high‐throughput screening of Cu‐based bimetallic alloys, offering a balanced combination of efficiency and predictive reliability in identifying promising candidates for CORR.

### 3.2 Feature Sensitivity Analysis

**FIGURE 5:**
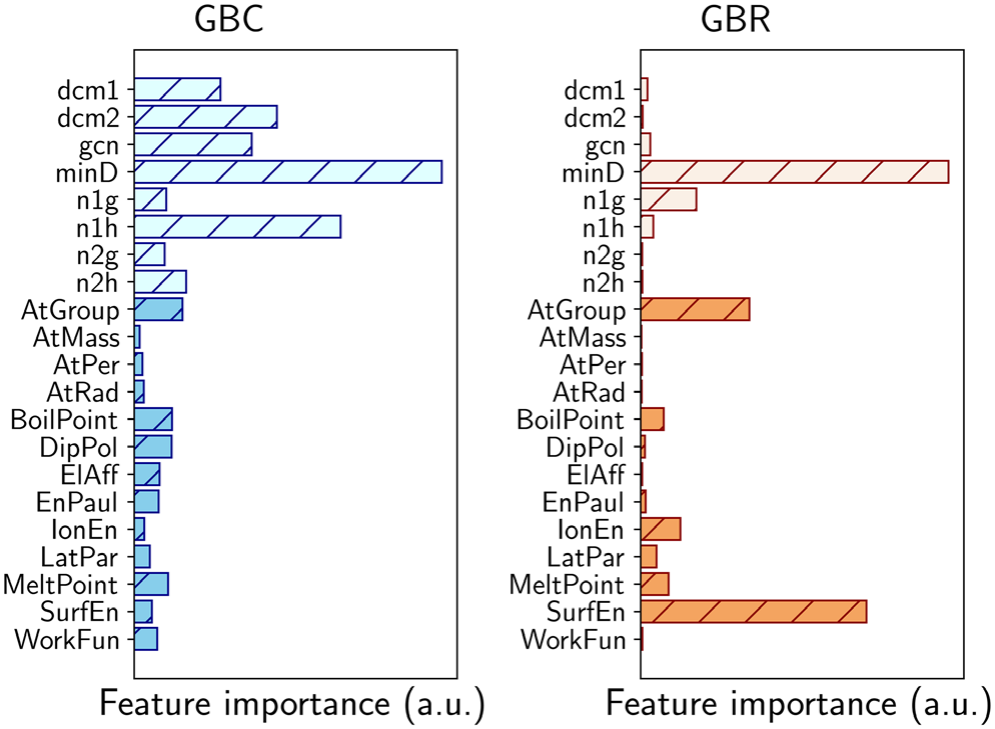
Feature sensitivity analysis obtained for the GBC (left) and GBR (right). Feature definitions are provided in the Method section. Lighter bars represent geometrical features, while darker bars indicate chemical features (blue for classification and orange for regression).

To gain further insight into the decision‐making process of the ML models, we performed a feature sensitivity analysis for both the classification and regression tasks. In Figure 5, we report the feature sensitivity analysis obtained for the GBC (left panel) and GBR (right panel) models. Geometric features are depicted in light blue (GBC) and light orange (GBR), while chemical features are depicted in blue and orange, respectively. For the classification task, the most relevant descriptors are those characterizing the local environment of the adsorption site. In particular, minD (the minimum distance between the binding site and the guest atom) emerges as the dominant feature, in line with observations from our previous study [55]. In addition, the GCN of the adsorption site and the local symmetry of the alloying‐atom arrangement, captured by the dcm1, dcm2 descriptors, and (indirectly) by the number of neighboring Cu atoms (n1h) also significantly contribute to model predictions. On the other hand, the impact of chemical descriptors is less straightforward. No individual chemical feature clearly dominates; instead, their contribution appears to result from a collective effect. Regarding the prediction of $\Delta E_{\mathrm{CO}}$ values, we note that among the geometric features, minD remains the most important. In contrast, the remaining descriptors of the local environment have a much smaller impact on model predictions. Chemical descriptors also play a significant role in this case: in particular, the atomic group of the alloying atom and its surface energy (referred to the hexagonal crystalline surface of the guest species) are identified as key contributors to model performance. The latter, in particular, aligns with physical expectations, as higher surface reactivity is generally associated with stronger molecular binding. These observations suggest that, while the geometric configuration, especially the proximity to alloying atoms, plays a dominant role in both classification and regression tasks, chemical features, particularly in the classification case, contribute in a more collective manner. While the use of more advanced descriptors, such as electronic structure parameters like the d‐band center, could potentially offer clearer, one‐to‐one correlations with adsorption site stability, they typically require prior DFT calculations. In this work, we intentionally selected a physically interpretable set of features based solely on structural descriptors and tabulated elemental properties. Despite this simplification, the resulting models achieved high predictive accuracy, underscoring the strength and practicality of our data‐efficient approach.

### 3.3 Identification of CuM Alloy Surfaces Favorable for CO Dimerization

Now that CO adsorption energies can be predicted reliably across a wide range of Cu‐based bimetallic surfaces, we apply the developed framework to identify alloy compositions expected to promote CO dimerization more effectively than pure Cu, thereby enhancing the likelihood of forming C_2_ products during CO_2_ electroreduction. We report in Figure S2 a graphical scheme of the adopted workflow. It is well established that Cu(100) is significantly more active than Cu(111) in promoting the formation of C_2_ and higher‐order carbon products [18]. Accordingly, our analysis is restricted to the (100) surface, with future work planned to extend the investigation to Cu(111). Previous studies [58, 59, 60] have shown that CO dimerization on Cu(100) occurs when two CO molecules are adsorbed on facing bridge sites. This configuration favors C–C bond formation, with the resulting dimer placed on the hollow site in between the two bridge sites. The reaction energy for CO dimerization on pure Cu(100), calculated by Wang et al. at constant applied potential U=−1.1 V vs SHE [90], is 0.16 eV and is used here as a reference to evaluate the performance of CuM alloys. We consider a Cu‐based alloy to be a promising candidate for C_2_ product formation if it shows a more exothermic CO‐dimerization reaction and a lower activation energy than pure Cu. Inspired by the trends reported in that same study, where a small set of bimetallic systems showed more favorable dimerization energetics, we defined a simple set of screening criteria aimed at identifying alloy surfaces where C–C coupling may be more favorable than on pure Cu(100). Focusing on pairs of opposite bridge sites, a configuration was considered favorable for CO dimerization if the combined adsorption energies of the two bridge sites, labeled a and b, satisfied the condition

(1) $$\Delta E_{\mathrm{CO}}^{a}+\Delta E_{\mathrm{CO}}^{b}\;>\;2\Delta E_{\mathrm{CO}}^{B}$$

where $\Delta E_{\mathrm{CO}}^{B}$ is the CO adsorption energy on a pristine Cu(100) bridge site. In cases where one of the two adsorption sites was classified as unstable, the configuration was still retained as promising if the stable site alone fulfilled the criterion

(2) $$\Delta E_{\mathrm{CO}}^{a/b}\;>\;2\Delta E_{\mathrm{CO}}^{B}$$

These two conditions serve as a simple and practical guideline to identify surface configurations that may favor CO dimerization more than pure Cu(100). However, we note that they are based on simple energetic arguments and do not account for other relevant factors, such as kinetic barriers or solvation effects. Further validation is therefore needed to assess their broader applicability across different alloy systems.

**FIGURE 6:**
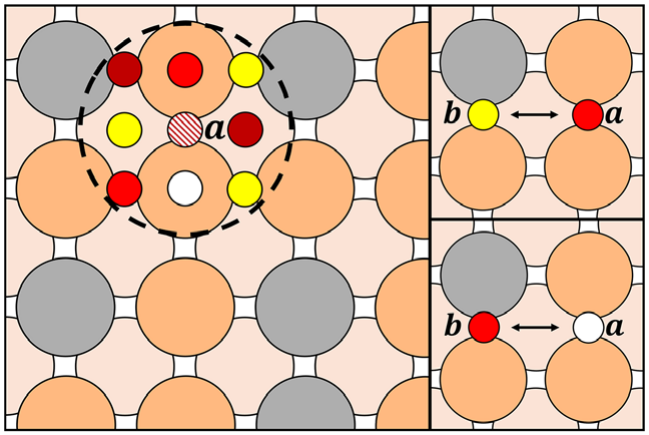
Example of structures considered in the screening process. Cu and M atoms are shown as orange and gray circles, respectively. Smaller circles represent the adsorption sites, with colors indicating the strength of CO adsorption: dark red for strong, red for moderate, and yellow for weak adsorption. White circles denote unstable sites. Site a is the one under analysis during the screening, and the dashed black line indicates its nearest neighbors. Site b indicates the bridge site facing a.

The screening was performed using GBC and GBR models to predict site stability and CO adsorption energy, respectively, on Cu_1−*x*_M_*x*_ (4×4) surface supercells. Four surface M‐atom concentrations were examined: x=0.063, 0.13, 0.19, and 0.25, corresponding to 1, 2, 3, and 4 substitutional atoms on the surface layer. The screened set of CuM catalysts was expanded beyond the original 15 systems used in model training to include 14 additional elements (Ru, W, Zn, Ga, In, Cr, Mn, Sc, Y, Zr, Rh, Ta, Re, and Os) resulting in a total of 29 guest species. Each CuM system was sampled using 49 inequivalent, randomly generated surface configurations: 1 for x=0.063, 5 for x=0.13, 10 for x=0.19, and 33 for x=0.25. To ensure consistency when comparing the effect of different alloying atoms on CO adsorption, the same set of configurations was used across all alloy systems. Once the CO adsorption energies were predicted for all binding sites across the CuM alloys and configurations, we applied the screening strategy described in Figure 6, which relies on the two conditions introduced earlier (Equations (1) and (2)). In the illustrative example shown in Figure 6, large circles represent Cu (orange) and M (gray) surface atoms, while smaller circles denote adsorption sites, color‐coded by their predicted CO adsorption energies: dark red for strong adsorption sites ($\Delta E_{\mathrm{CO}}\leq -1.1$ eV), red for moderate adsorption ($-1.1<\Delta E_{\mathrm{CO}}\leq -0.6$ eV), and yellow for weak adsorption sites ($-0.6<\Delta E_{\mathrm{CO}}<0$ eV). White circles indicate sites predicted to be unstable. These patterns are schematic, not based on actual ML outputs, and intended solely to clarify the screening procedure. For a given bridge site a, we first evaluated its closest neighboring sites (those within the black dashed circle in Figure 6, left panel). If none of the neighboring sites showed very negative $\Delta E_{\mathrm{CO}}$, which could prevent CO adsorption on a, we then analyzed the CO adsorption energy on the bridge site b, opposite to a. A pair was considered promising for CO dimerization if it satisfied one of the two conditions reported in (1) (Figure 6, upper right panel) and (2) (Figure 6, bottom right panel). We classified a CuM system as promising if at least 40 out of the 49 sampled configurations contained favorable bridge site pairs. According to this criterion, CuAg, CuAl, CuAu, CuZn, CuGa, and CuIn emerged as the most promising candidates.

**FIGURE 7:**
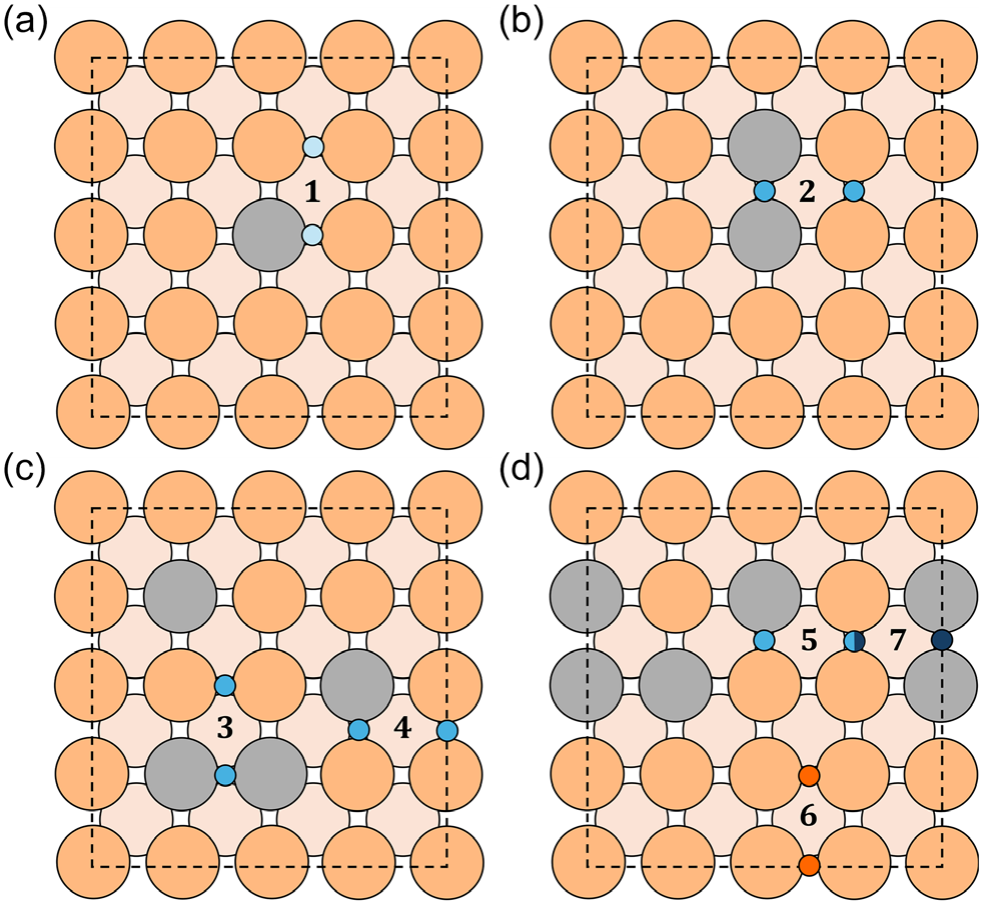
Selected CuGa surface structures used for testing. Three different surface M‐atom concentrations were considered: x=0.063 (a), x=0.13 (b), and x=0.25 (c,d). Large circles represent Cu (orange) and Ga (gray) atoms, while small circles mark the initial positions of adsorbed CO molecules. The color of each small circle indicates the calculated CO dimerization reaction energy: light red for ΔΩ>0 (endothermic), and blue to dark blue for ΔΩ<0 (exothermic), with darker shades corresponding to more favorable energetics. A half‐blue, half‐dark‐blue diamond indicates a CO adsorption site that participates in two distinct dimerization pathways.

**FIGURE 8:**
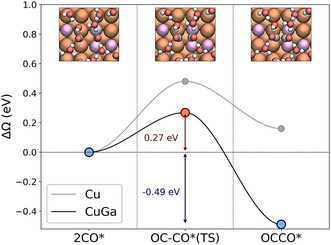
CO dimerization energy profiles on pristine Cu(100) (gray curve) and Cu0.75Ga0.25(100) (black curve) at –1.1 V vs SHE with an explicit water layer. The three top panels show the adsorbed CO pair at the initial, transition, and final states (water molecules partially cut for clarity). Atoms are colored: H (white), C (dark gray), O (red), Cu (orange), Ga (light purple). Reference activation barrier and reaction energy for pure Cu(100) are from Ref. [90].

To validate our screening results, we focused on CuGa as a representative alloy and explicitly computed the grand potential ΔΩ for CO dimerization at constant applied potential U=−1.1 V vs SHE, using configurations identified as promising by the ML‐based screening. This applied potential value was chosen since it is known to maximize C_2_ product formation during CO_2_RR (in particular, toward ethylene [91]) and matches that used by Wang et al., enabling a fair comparison. We did not consider alternative pathways to C_2_ products involving CHO or COH intermediates, as these routes are generally favored only under more reducing conditions [92, 93]. Figure 7 shows four of the tested configurations: (a) x=0.063, (b) x=0.13, and (c)/(d) x=0.25. Small circles mark the initial adsorption sites of the two CO molecules prior to dimerization. Each pair of CO binding sites is labeled 1–7 for clarity. The color indicates the corresponding reaction energy: light red for endothermic reaction (ΔΩ>0) and a gradient from light blue to dark blue for exothermic ones (ΔΩ<0), with darker shades representing more favorable (i.e., more negative) energies. The half–blue, half–dark blue circle indicates that adsorption sites involved in two distinct dimerization processes (one with the neighboring bridge site on the left, and one on the right). The calculated reaction energies and the reference value for pristine Cu [90] (highlighted in gray) are reported in Table 4. All tested CuGa structures, except configuration 6, exhibited pairs of bridge sites that lead to exothermic CO dimerization, with pairs 2, 3, 5, and 7 showing particularly favorable values. These findings suggest that alloying copper with Ga would enhance C–C coupling activity and confirm both the screening criteria and ML predictions. While the majority of configurations with negative reaction energies corresponded to sites identified as promising by our screening, a few false positives were observed. These discrepancies do not stem from limitations of the ML models themselves but rather reflect the need to refine the criteria in Equations (1) and (2) to better account for CO–CO interactions under higher coverage scenarios. Therefore, despite some exceptions, the screening protocol demonstrated good overall performance, accurately identifying favorable adsorption sites for a variety of surface configurations of the alloying atoms. Next, we focused on the most favorable pair identified in Figure 7, pair 7, and computed the complete reaction energy profile, from initial state through transition state to final dimer configuration. The key geometries along the CO dimerization pathway are depicted in the top panels of Figure 8: the initial configuration with two CO molecules arranged as in pair 7 (left), the transition state (center), and the resulting C–C bonded dimer (right). The calculated activation barrier from this pathway is 0.27 eV (indicated by the red arrow in Figure 8), significantly lower than that of pure Cu (0.48 eV, gray line in Figure 8) and that of an isolated Ga atom (Figure 7a), which is 0.43 eV [90]. This progressive reduction in activation energy with increasing Ga concentration highlights the potential of Ga alloying to enhance CO dimerization, a key step toward C_2_ product formation, thereby making the reaction pathway more favorable than on pristine Cu. Moreover, previous experimental studies have demonstrated the feasibility of synthesizing Cu–Ga surface alloys, further supporting the practical relevance of these findings [28].

**TABLE 4:** Reaction energies for CO dimerization on the bridge‐site pairs highlighted in Figure 7. (a)–(d) correspond to the panels depicted in Figure 7. The benchmark value for pristine Cu [90] is shaded in gray.

Structure	Bridge pair	Reaction energy, eV
Cu(100)	Any	0.16
(a)	1	−0.10
(b)	2	−0.36
(c)	3	−0.33
	4	−0.24
(d)	5	−0.37
	6	0.10
	7	−0.49

### 3.4 Analysis of the Selected CuM Surfaces

**FIGURE 9:**
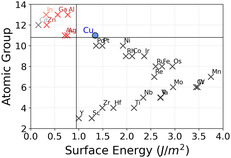
Scatter plot of atomic group versus surface energy for all materials considered in the screening process. The promising alloying elements (red crosses) occupy a chemically well‐defined region, clearly separated from the rest. The presence of a nonpromising element within this region (Cd) indicates that the stability of the adsorption sites must also be considered.

**FIGURE 10:**
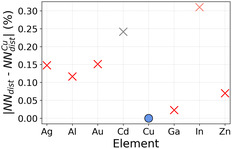
Percentage difference of the average interatomic distances in the stable crystalline phase of each pure element with respect to those of pure Cu.

As discussed in the previous sections, we have focused on the CO adsorption energy as the key descriptor determining whether C_2_ products can form. This choice reflects the central role of CO binding strength in balancing surface stabilization and C–C coupling. For this reason, we begin by examining the chemical features that most strongly influence the CO adsorption energy predictions, as identified by the ML feature sensitivity analysis (right panel of Figure 5). Among the available descriptors, the atomic group and the surface energy of the alloying element emerge as the most relevant. Interestingly, the 2D map reported in Figure 9 shows that the promising alloying elements (highlighted by red crosses) cluster within a well‐defined and compact chemical region, clearly separated from the rest of the metals considered. This clustering indicates that enhanced CO–CO coupling is associated with specific intrinsic properties of the guest elements rather than being accidental. In particular, we observe that alloying copper with elements having a surface energy slightly lower than that of Cu (blue circles) favors CO dimerization. This trend is fully consistent with our previous study [55], where we demonstrated a linear relationship between the surface energy of the alloying metal and the CO adsorption energy (in proximity of the guest species): metals with higher surface energy are generally more reactive and therefore bind CO more strongly. Hence, according to the Sabatier principle, introducing a metal with a slightly lower surface energy than Cu locally weakens the CO adsorption, shifting the binding strength away from over‐stabilization and toward a regime more favorable for CO–CO coupling. Regarding the atomic group, we note that the alloying elements falling within the promising chemical region belong to groups with fully filled outer d orbitals (with the exception of Pd, which lies outside this region). This observation provides an additional chemical rationale for the observed trend and is consistent with our previous work [55], where we showed that elements with AtGroup ≥11 tend to induce weaker CO adsorption. Together, surface energy and atomic group coherently support the Sabatier‐based interpretation introduced above. Nevertheless, chemical trends alone are not sufficient to determine catalytic performance, and adsorption site stability plays a crucial role in determining whether a given alloy can effectively promote CO–CO coupling. Indeed, within the promising chemical region, we identify two elements—Cd and In—that cannot host CO on Top sites. However, despite this apparent similarity, their impact on C–C coupling is markedly different: In emerges as a good candidate, whereas Cd does not. This difference arises because In moderately perturbs CO adsorption on neighboring Cu sites, thereby preserving stable adsorption configurations that enable CO–CO coupling. In contrast, Cd induces a stronger destabilization of CO adsorption, preventing CO from binding on adjacent sites and thus suppressing further reaction steps. To further clarify this behavior, we consider the percentage difference in the average interatomic distance ($NN_{dist}$) of the stable bulk phases of Ag, Al, Au, Cd, Ga, In, and Zn relative to Cu (Figure 10, data extracted from the Materials Project website [69]). While both Cd and In exhibit a large lattice mismatch with Cu, their effects on CO adsorption are qualitatively different, indicating that geometric mismatch alone cannot account for the observed trends. A comparison of additional chemical properties helps rationalize this discrepancy. In particular, the boiling and melting points of Cd are significantly lower than those of In, suggesting weaker metallic bonding in Cd. This weaker cohesion likely amplifies the destabilizing effect exerted by Cd on nearby adsorption sites, as also supported by the relevance of these features in the classification task (left panel of Figure 5). In summary, the ability of CuM alloys to promote CO–CO coupling arises from a subtle interplay between chemical and structural effects. Alloying elements with slightly lower surface energy than Cu and fully filled outer d orbitals tune the local CO adsorption strength toward the optimal Sabatier regime. At the same time, the metallic bonding strength and the bulk interatomic distances of the guest element modulate the stability of neighboring adsorption sites, either enabling or suppressing CO dimerization pathways. Overall, these results show that while general chemical and physical principles provide valuable guidance, their combined effects are highly nontrivial. ML is therefore essential, as it captures these complex correlations and enables accurate, scalable, and reliable high‐throughput screening of Cu‐based bimetallic catalysts.

## 4 Conclusion

In this work, we developed a machine learning framework to identify Cu‐based bimetallic surfaces that can promote CO dimerization, a key step in the formation of multicarbon products during the electrochemical reduction of CO_2_. The approach combines classification and regression models trained on a DFT dataset, using simple and physically interpretable features to predict CO adsorption behavior across a wide range of alloy compositions and surface configurations (approximately 91,000 adsorption sites). In parallel, we introduced a set of geometric and energetic screening criteria to identify adsorption site pairs favorable for CO–CO coupling, enabling a targeted and interpretable selection of promising candidates.

We applied this workflow to 29 CuM alloys, focusing on the (100) facet, widely recognized for its ability to promote CO–CO dimerization. By analyzing the CO adsorption behavior on over 91,000 screened adsorption sites, we identified six alloys (CuAg, CuAl, CuAu, CuZn, CuGa, and CuIn) featuring a high density of sites favorable for CO dimerization. Among these, CuGa was chosen for detailed validation due to its limited prior exploration in CO_2_RR and its strong performance revealed by our screening. Constant‐potential DFT calculations confirmed the machine learning predictions, showing that CO dimerization on CuGa(100) proceeds with both reaction energy and activation barrier at least 0.2 eV lower than on pure Cu(100). To conclude, building on the feature sensitivity analysis provided by our machine learning models, we were able to rationalize, in terms of chemical and physical properties, why certain Cu‐based alloys emerged as the most promising candidates for CO dimerization.

From a methodological standpoint, this study demonstrates that the proposed ML‐based workflow provides reliable and computationally efficient predictions, exploiting simple criteria for the screening process based on energetic considerations. From a materials discovery perspective, the results highlight the potential of CuGa and related alloys to enhance CO dimerization, pointing toward possible further optimization strategies through systematic tuning of alloy composition and surface structure to improve selectivity and activity.

## Supporting Information

Additional supporting information can be found online in the Supporting Information Section. **Supporting Fig. S1**: Starting geometry of two CO molecules adsorbed on a CuGa surface, before dimerization occurs. The explicit water layer contains 10 H_2_O molecules. Color code: orange and pink for Cu and Ga atoms, respectively; gray, red, and white for C, O, and H atoms. **Supporting Fig. S2**: Graphical scheme of the adopted workflow. **Supporting Fig. S3**: Geometrical features considered in the dataset. Left panel: minD, n1h, n1g, n2h, and n2g. Right panel: dcm1 and dcm2, defined as the distances between the binding site and the centers of mass of the impurities within the first (blue cross) and second (red cross) neighboring shells, respectively. **Supporting Fig. S4**: Structures considered in the TsC set. Smaller circles depict the inequivalent binding sites used to test the ML models: yellow for top sites, blue for bridge sites, and red for hollow sites. **Supporting Fig. S5**: Structures considered in the TsG set. Smaller circles depict the inequivalent binding sites used to test the ML models, with the same color scheme previously introduced.

## Funding

This study was supported by MSCA‐DN (Grant 101072830).

## Conflict of Interest

The authors declare no conflicts of interest.

## Supporting information

Supplementary Material

## Data Availability

The data that support the findings of this study are available from the corresponding author upon reasonable request.
