# Supplementary material for: A Machine Learning‐Guided Search of Cu‐Based Bimetallic Alloys That Favor CO Dimerization in CORR
Source: ChemSusChem. 2026 Mar 26;19(6):e202501603. doi: 10.1002/cssc.202501603 (PMC13021306; doi:10.1002/cssc.202501603)
Supplement: Supplementary file 1 — Supplementary Material [file CSSC-19-e202501603-s001.pdf]

Supporting Information: A machine learning-guided search of Cu-based bimetallic alloys that favor CO dimerization in CO_2RR

Mattia Salomone, Wei Wang, Federico Raffone, Michele Re Fiorentin, Francesca Risplendi, and Giancarlo Cicero*

Department of Applied Science and Technology, Politecnico di Torino, Italy

E-mail: giancarlo.cicero@polito.it

Table of Contents

1. Explicit water layer structure S2
2. Definition of Geometrical Features S2
3. Description of the Test sets S3
4. References S4

1. Explicit water layer structure

We report in Fig. S1 one of the initial configurations used to calculate the CO dimerization reaction energy. The geometry of the explicit water layer consists of 10 H_2O molecules positioned in an ice-like structure.[S1]

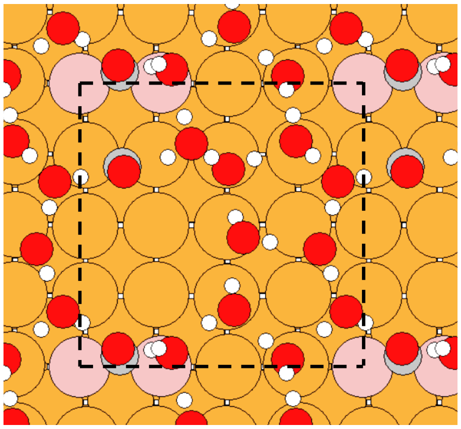

Figure S1: Starting geometry of two CO molecules adsorbed on a CuGa surface, before dimerization occurs. The explicit water layer contains 10 H_2O molecules. Color code: orange and pink for Cu and Ga atoms, respectively; gray, red, and white for C, O, and H atoms.

2. Workflow

We report in Fig. S2 a schematic representation of the workflow adopted in this study. First, two distinct datasets were constructed: one containing 1,515 data points describing site stability (used to train the classification model), and another containing 601 data points describing the CO adsorption energy on the stable sites (used for regression). The trained ML models were then applied in a high-throughput screening of the CO adsorption behavior across more than 90,000 adsorption sites for stability assessment and approximately 50,000 sites for adsorption energy prediction. Finally, the most promising adsorption sites for CO dimerization were identified by analyzing the ML-predicted adsorption stabilities and energies.

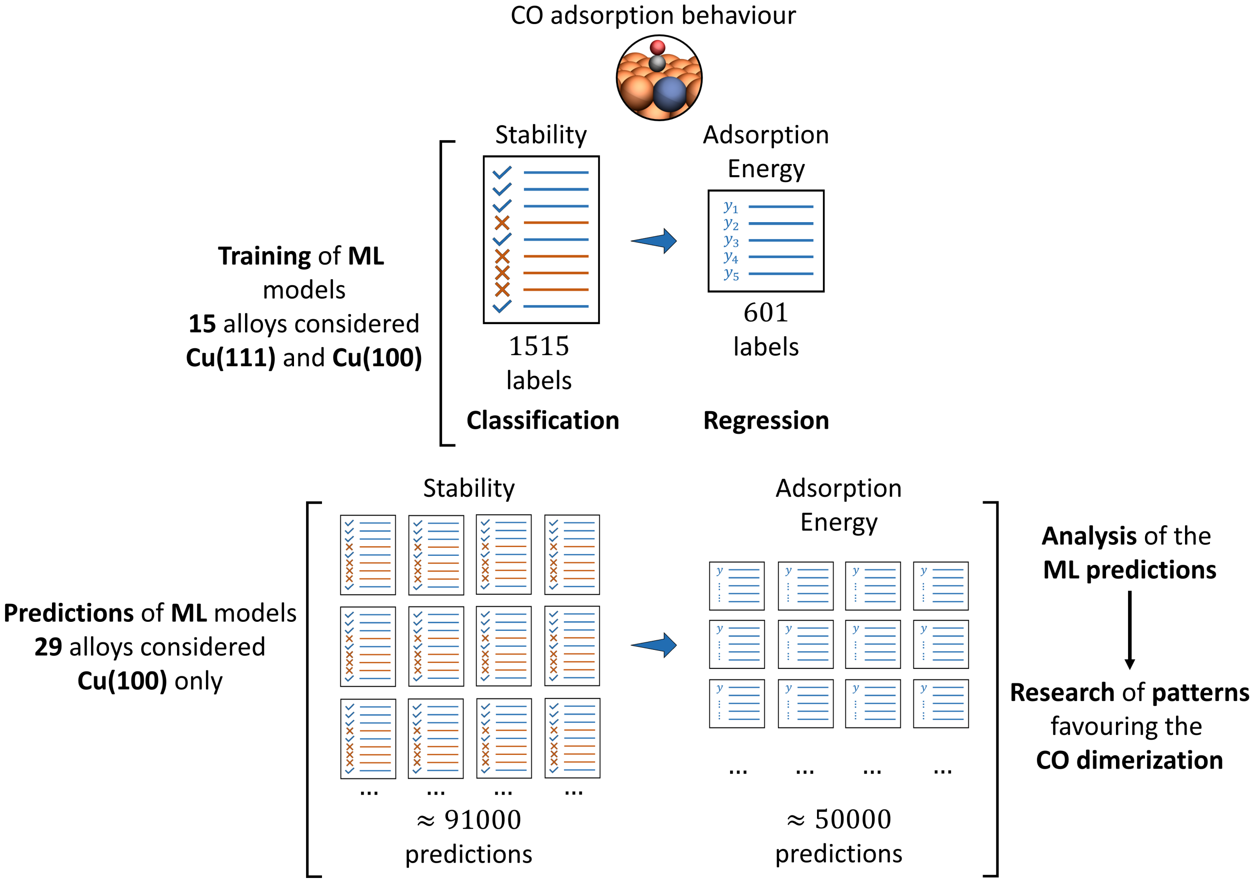

Figure S2: Graphical scheme of the adopted workflow.

3. Definition of Geometrical Features

In addition to the Generalized Coordination Number[S2] and the minimum distance between the binding site and a nearby impurity, we considered six geometrical features to describe the local environment of the CO binding site. These include the number of Cu and guest atoms within the first and second nearest-neighbor shells—indicated by the blue and red dashed lines in Fig. S2 (left panel), respectively—as well as the distance from the binding site to the centers of mass of the impurity atoms located within those same shells (blue and red crosses in Fig. S2, right panel).

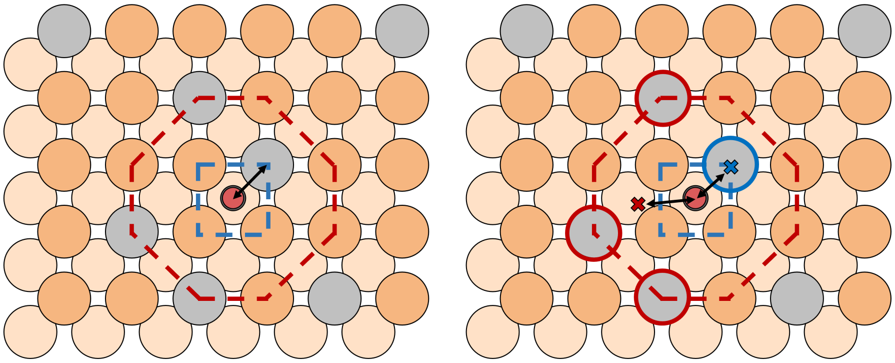

Figure S3: Geometrical features considered in the dataset. Left panel: minD, n1h, n1g, n2h, and n2g. Right panel: dcm1 and dcm2, defined as the distances between the binding site and the centers of mass of the impurities within the first (blue cross) and second (red cross) neighboring shells, respectively.

4. Description of the Test sets

Structures used for the two test sets, namely TsC (Fig. S3) and TsG (Fig. S4). The first test set (TsC) is designed to evaluate the predictive capabilities of the ML models on impurity elements not included in the training set. Although the geometries in TsC slightly differ from those used during training, we bench-marked the model performance on impurity elements present in the training set but applied to these new geometries. The results showed that the predictions for known elements remained consistent with those obtained on training geometries, suggesting that such structural variations do not significantly impact the predictions. Conversely, TsG is intended to assess the generalization ability on entirely new surface geometries.

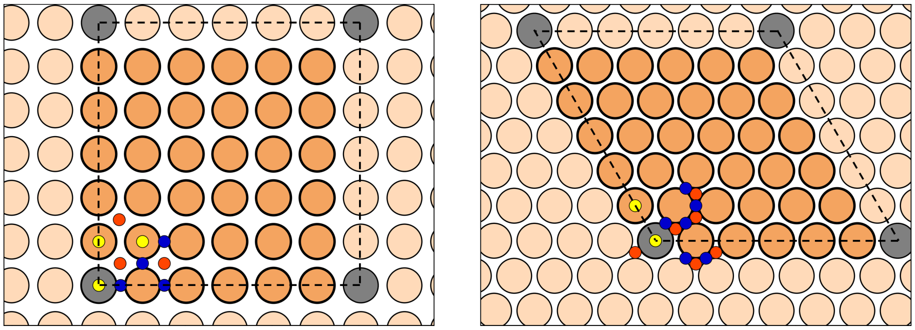

Figure S4: Structures considered in the TsC set. Smaller circles depict the inequivalent binding sites used to test the ML models: yellow for top sites, blue for bridge sites, and red for hollow sites.

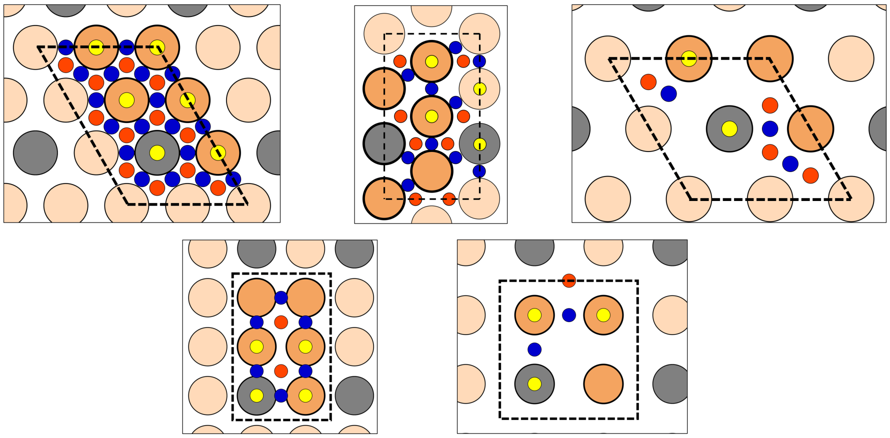

Figure S5: Structures considered in the TsG set. Smaller circles depict the inequivalent binding sites used to test the ML models, with the same color scheme previously introduced.

References

(S1) Montoya, J. H.; Shi, C.; Chan, K.; Nørskov, J. K. Theoretical Insights into a CO Dimerization Mechanism in CO2 Electroreduction. *The Journal of Physical Chemistry Letters* **2015**, *6*, 2032–2037, PMID: 26266498.

(S2) Calle-Vallejo, F.; Bandarenka, A. S. Enabling Generalized Coordination Numbers to Describe Strain Effects. *ChemSusChem* **2018**, *11*, 1824–1828.
